# Heterogeneous impact of mask mandates on U.S. masking behavior: an interrupted time series study

**DOI:** 10.1093/aje/kwaf236

**Published:** 2025-10-22

**Authors:** Benjamin Rader, Christina M Astley, Laura F White, John S Brownstein, Matthew P Fox

**Affiliations:** Department of Epidemiology, Boston University School of Public Health, Boston, MA, United States; Innovation and Digital Health Accelerator, Boston Children’s Hospital, Boston, MA, United States; Department of Anesthesiology, Harvard Medical School, Boston, MA, United States; Innovation and Digital Health Accelerator, Boston Children’s Hospital, Boston, MA, United States; Division of Endocrinology, Boston Children’s Hospital, Boston, MA, United States; Department of Pediatrics, Harvard Medical School, Boston, MA, United States; Department of Biostatistics, Boston University School of Public Health, Boston, MA, United States; Innovation and Digital Health Accelerator, Boston Children’s Hospital, Boston, MA, United States; Department of Pediatrics, Harvard Medical School, Boston, MA, United States; Department of Epidemiology, Boston University School of Public Health, Boston, MA, United States; Department of Global Health, Boston University School of Public Health, Boston, MA, United States

**Keywords:** masking, adherence, interrupted time series, mandates, ceiling effect, policy evaluation, participatory surveillance, nonpharmaceutical interventions

## Abstract

Despite widespread implementation of mask mandates for COVID-19 transmission control, studies examining their effectiveness have yielded mixed results ranging from strong benefits to no effect. These inconsistencies may arise from a variety of methodological and measurement challenges, including the implicit assumption that mandates truly modify masking behavior—the essential mechanism for transmission interruption. Here, we leverage self-reported mask adherence data from >34 000 individuals collected via a digital participatory surveillance platform between June 2, 2020, and January 1, 2021, to examine this assumption. Using an interrupted time series approach, we aggregate masking observations at the county level to analyze the effect of mandates on masking uptake across 555 diverse U.S. counties. We evaluate masking during the 14 days premandate and postmandate issuance, finding a modest 1-3 percentage point overall increases in masking. However, substantial heterogeneity was observed, with larger changes seen in counties initially exhibiting low mask adherence, the U.S. West, and on masking uptake in public compared to private settings. Our findings suggest that conflicting estimates of the effect of mandates on transmission reduction may reflect modification by heterogeneity in the mandates’ alteration of masking behavior. Future interventions should tailor mandates to local context and baseline adherence for maximal behavioral change.

## Introduction

Although the usage of face masks has become a common practice across the United States during the COVID-19 pandemic,^1^ their effectiveness in reducing transmission events remains a topic of debate.^2^^,^^3^ An important source of conflict over their usage stems from binding mask mandates often accompanying masking recommendations, perceived by many as an infringement on civil liberties.^3^^,^^4^ While there is robust evidence that masks can reduce respiratory disease spread,^5^^-^^8^ the impact of mask mandates on this reduction is less clear.^5^^,^^9^^-^^14^ This disconnect may be attributed to a variety of factors, including variable adherence to mandates,^5^^,^^13^ the occurrence of substantial disease spread in private settings—despite public mandate compliance,^15^ aggregation bias obscuring that masking is protective in some settings and not in others, and the inherent difficulty of accurately measuring the effects of complex government policies.^16^ These divergent findings can also be considered within the historical context of inconsistent compliance to public health mandates,^17^ despite clear evidence of the effectiveness of the mandated behavior (eg, seat belt wearing).^18^

Accurately measuring the effect of mask mandates on disease outcomes presents several challenges, including confounding by indication. Mask mandates are typically introduced in response to a surge in COVID-19 incidence, which often simultaneously triggers other mitigation measures and behavioral changes that can confound the isolation of the mandates’ effects. Consequently, studies employing pre–post designs might capture trends in alternative behavioral responses to disease dynamics (eg, social isolation due to perception of increased risk) rather than the effect of the mandates on outcomes mediated through the desired masking behavior. If masking is increasing due to exogenous factors at the same time mandates are issued, studies that do not account for this will likely overstate the causal effect of mandates themselves. This potential confounding may also explain why some studies have found no added benefit of mask mandates when accompanied by other nonpharmaceutical interventions,^10^ while others found a reduction in COVID-19 mortality implausibly just one week following their issuance.^11^ Moreover, mandates were not introduced at random: they were often enacted in jurisdictions where public support for such policies was already high, and at times when citizens were already voluntarily adopting them. In this sense, mandates may partly represent an ex-post validation of behaviors communities were already participating in, which complicates attempts to isolate their independent effect.

In addition to the complexities of establishing a causal link between mandates and disease outcomes, another possible reason for the inconsistent results between the impacts of masking and mandates might stem from the variability in mask adherence due to mandates. Policies spanning multiple jurisdictions and the potential for geographic spillover effects can substantially influence this variability. Mandates have been implemented at different levels of government and geography, with some local mandates being issued before broader statewide ones (eg, Cuyahoga County, Ohio, the location of Cleveland, implemented a mandate approximately 2 weeks prior to Ohio State).^19^ Early county-level mandates may effectively increase adherence but obscure the impact of later statewide ones because they had already caused the population proportion masking to peak. Additionally, geographic spillover effects can occur when neighboring regions respond to mandates in adjacent localities (eg, Akron, Ohio masking levels being influenced by Cleveland’s mask mandate^20^) before being formally required to do so. Previous studies measuring the effect of mask mandates on adherence to masking have primarily focused on statewide mandates and mask usage aggregated at the state level, which may be downwardly biased by these geographic spillover effects and obscure important heterogeneities in the intervention’s impact. This hypothesis is supported by a U.S. study that found increased mask-wearing reduced COVID-19 transmission, but statewide mask mandates did not alter mask-wearing.^5^

To better understand the disease mitigation benefits of masking and mask mandates, we sought to answer a fundamental question: do mask mandates modify masking behavior? Importantly, because mask usage forms a critical link in the causal chain from mandates to disease mitigation, if mandates do not alter mask-wearing behavior, they are unlikely to influence the control of disease transmission. To mitigate the issues arising from multi-jurisdictional mandates and geographic spillover effects, we conduct our analysis at the county level. Using data from the first year of the COVID-19 pandemic, we estimate how much initial implementations of county-level government mask mandates changed self-reported masking behavior using an interrupted time series (ITS) analysis across the United States. By examining the effect of mask mandates through a more local lens, we aim to understand the heterogeneity of the intervention’s effectiveness across different communities, which is crucial for informing future infectious disease mitigation strategies.

## Methods

First, we measured the effect of county-level mask mandates on self-reported masking behavior during the initial phase of the COVID-19 pandemic using an ITS stacked design. Next, we ran subgroup analyses stratified by geography, urbanicity, and population proportion self-reporting masking prior to the issuance of mandates. Finally, we employ a pooled ITS design to ensure our results are robust to model specification.

### County mask mandate (exposure)

We extracted dates of county mandates from “Tracking Mask Mandates During the Covid-19 Pandemic,” a database of county- and state-level mask interventions^19^ previously used to understand adherence to mask mandates at the state level.^14^ All U.S. counties (*n* = 624 counties in 14 U.S. states) that issued a county-level mask mandate after June 17, 2020 (14 days after the start of mask adherence data, described below) preceding, concurrent with, or in the absence of respective state mandate are included for analysis.

### Self-reported mask adherence (outcome)

Individual self-reports of mask-wearing in public and private settings were collected between June 2, 2020, and January 1, 2021, by the OutbreaksNearMe anonymous serial cross-sectional web survey (*N* > 3 000 000) in the United States.^5^^,^^21^ Survey respondents were asked multiple demographic and behavioral questions, including their home location and how likely they were to wear a mask “while grocery shopping” (masking in public settings) or “while visiting with family or friends in their homes” (masking in private settings) on a four-point scale. Individuals are considered masked if they respond that they are very likely to wear a mask and not masked if they report they are somewhat likely, not so likely to mask or not likely at all.

For each county in the dataset, masking was measured for each of the 14 days before and after the issuance of mandates to assess the short-term effect of mask mandates. Measuring masking for 14 days postmandate was chosen because this time period was within 2-3 generation intervals of early COVID-19 variants (ie, within the window of time necessary to acutely modify a circulating wave’s dynamics).^22^

The OutbreaksNearMe survey is a collaboration between Boston Children’s Hospital and Momentive.ai. The survey employs a nonprobability sampling method known as river sampling to gain a diverse representation of U.S. adult respondents. The potential participant pool, or the sampling frame, includes all adults in the United States who are using the SurveyMonkey platform for alternative activities. When these individuals have completed their other activities on the platform, they are then randomly invited to participate in the OutbreaksNearMe Survey (approximately 11% presented with the option to take the survey to complete it). Survey participation was IP-restricted to one response per household. As the SurveyMonkey user base is highly diverse, this method results in a varied sample for the OutbreaksNearMe survey. Additionally, inverse probability weights derived from the 2019 U.S. census demographics (age, race, sex, education, and geography) are applied to generalize the survey to the broader United States. This weighting scheme generates a pseudo-population, henceforth referred to as the sample population. Respondents were not incentivized to complete the survey and individuals younger than 13 years old or older than 100 years old were excluded. Previous validation efforts have shown the OutbreaksNearMe population to be of similar composition to the United States according to census demographics and CDC disease estimates.^21^

The OutbreaksNearMe survey was approved by the Boston Children’s Hospital IRB and received a waiver of informed consent.

### Analysis

We used an ITS stacked design^23^ to estimate changes in masking in the 14-day period following a county-level mandate compared to the 14 days preceding the mandate. This design utilized individual-level masking observations (binary: self-reported masked vs not), $i$, as rows (ie, a panel design) within a single regression framework. When estimating the effects of mandates in the entire population, the dataset for analysis consisted of the entire study sample. The dataset for analysis was then filtered down to smaller samples (eg, only individuals living in urban areas) when estimating the heterogeneity of the effect (more details in *Analysis of heterogeneity* below). The stacked ITS model was based on a modified Poisson regression model with a log link function and robust error variance^24^ and was fit to assess if there is a change in slope and intercept of masking in $s$ setting (public [measured by grocery shopping masking behavior] versus private [measured by masking behavior with family/friends]) following the introduction of a mandate:


$$ \mathit{\ln}\left[p{(Masking)}_{s,i}\right]={\beta}_0+{\beta}_1 Tim{e}_i+{\beta}_2 Mandat{e}_i+{\beta}_3 TimeSinceMandat{e}_i, $$

where $Masking$ is a binary variable indicating if the individual self-reported masking, $Time$ is an ordinal variable measured in days from 0 (first day of observation) to 28 (last day of observation), $Mandate$ is an indicator variable representing the presence of a mask mandate at the time of observation, and $TimeSinceMandate$ is an ordinal variable with a value of 0 for the premandate period and the first day of a mandate after which it increases up to 14 for each day a mandate is in place.

The ITS analysis estimates if a change in the presence of a mandate is associated with an immediate shift in masking levels (${\beta}_2$, known as a pulse effect or one-time change in masking) and/or change in slope (${\beta}_3$, known as a ramp effect or a change in masking trends). The model assumes that each individual’s choice to mask is binary, makes no distinction in the type of masks individuals are wearing, and assumes that the 14-day period prior to the mandate was sufficiently long to characterize it.^16^ The counterfactual that the effect is compared against assumes that the pre-intervention trend continued and that there were no exogenous influences on the measured outcome.

Robust standard errors were calculated with the *sandwich* (v3.0-2) and *lmtest* (v0.9-40) R packages. From the results of the ITS analysis, we computed the marginal effects^24^ of mask-wearing to estimate the average increase in mask-wearing in counties that implemented a mandate.

A modified Poisson regression with robust error variance was chosen as a primary model for our binary masking data. This decision was driven by our intent to report relative risk, which is more interpretable in the context of common outcomes such as mask-wearing.^25^

Analyses were conducted to assess the heterogeneous effect of mask mandates on mask-wearing. We focused on differences in the effect of mandates based on the counties they were implemented in. To do so, we stratified by county features: U.S. census region geography (Northeast, Midwest, South, West), urbanicity (metropolitan, micropolitan, and nonmetropolitan, defined by the National Center for Health Statistics Urban–Rural classification scheme), tertile of the previous degree of mask-wearing (measured by computing the tertile of each county based on the mean percentage of the population reporting masking in grocery stores and with family/friends pooled across the 14-days prior to mandate implementation), calendar time of mandate implementation (June vs July 2020), and whether or not an adjacent (ie, Queen contiguity) county had already issued a county mandate. These analyses were executed by filtering the dataset for analysis, $d$, down to the relevant counties and then recalculating the combined parameter coefficients for each stratum (eg, the effect of mandates in the Northeast U.S.).

### Sensitivity analysis of model specification

To ensure our results were robust to model specification and ITS parameterization, we conducted a sensitivity analysis utilizing a pooled design ITS model (Appendix S1). The pooled design fits separate ITS models for each county and pools parameter estimates to measure the effect of mandates.

## Results

### Study respondents

From June 2, 2020, to January 1, 2021, 34 106 unique respondents submitted self-reports of their masking behavior (Table 1). Respondents were 53.7% female, 45.1% male, and 1.2% transgender or nonbinary, compared to the 2019 U.S. American Community Survey that estimates the United States to be 50.8% female and 49.2% male.^26^ Self-reported household income in the lowest (<$30 000) and highest (≥$150 000) yearly categories contained 23.8% and 10.2% of the respondents compared to 23.6% and 14.4% estimated by the U.S. census, respectively. Self-reports of masking were not evenly distributed across demographics (Table 1); however, respondent makeup was similar for those survey before and after mandate implementation (Table S1).

**Table 1:** Self-reported demographics and masking behavior from U.S. adults (*n* = 34 106) who lived in U.S. counties (*n* = 555) that issued mask mandates preceding, concurrent with, or in the absence of respective state mandates.

**Characteristic**	**Weighted survey respondents total: 34 106** ***n* (%)**	**Survey respondents who were very likely to mask with family/friends total: 12 586 (36.8%)** ***n* (%)**	**Survey respondents who were very likely to mask while grocery shopping total: 28 362 (83.2%)** ***n* (%)**
Race			
White	26 066.5 (76.4)	7662.3 (64.3)	19 800.8 (74.2)
Black	4346.0 (12.7)	2682.7 (22.5)	3819.2 (14.3)
Hispanic	981.3 (2.9)	465.7 (3.9)	866.0 (3.2)
Other	1869.2 (5.5)	790.8 (6.6)	1585.4 (5.9)
Missing	843.1 (2.5)	316.4 (2.7)	611.0 (2.3)
Gender			
Female	18 312.8 (53.7)	6879.5 (57.7)	15 033.7 (56.3)
Male	15 383.2 (45.1)	4877.5 (40.9)	11 353.3 (42.5)
Transgender or nonbinary	410.1 (1.2)	160.9 (1.4)	295.4 (1.1)
Education			
High school or less	13 385.1 (39.2)	4732.3 (39.7)	9709.2 (36.4)
Some college	10 493.3 (30.8)	3476.2 (29.2)	8152.6 (30.6)
College or more	6446.8 (18.9)	2145.3 (18.0)	5404.0 (20.3)
Postgraduate degree	3780.9 (11.1)	1564.2 (13.1)	3416.6 (12.8)
Age, years			
18-29	6140.2 (18.0)	1849.9 (15.5)	4446.5 (16.7)
30-39	5732.1 (16.8)	1823.3 (15.3)	4138.8 (15.5)
40-49	5633.8 (16.5)	1714.5 (14.4)	4151.3 (15.6)
50-64	9190.4 (26.9)	3399.3 (28.5)	7409.2 (27.8)
65-74	5580.8 (16.4)	2348.6 (19.7)	4912.4 (18.4)
75+	1828.7 (5.4)	782.3 (6.6)	1624.2 (6.1)
Household income			
Less than $30 000	8112.1 (23.8)	3410.6 (28.6)	6204.8 (23.3)
$30 000-49 999	5439.7 (15.9)	1974.0 (16.6)	4261.6 (16.0)
$50 000-$74 999	5745.2 (16.8)	1848.0 (15.5)	4399.9 (16.5)
75 000-$99 999	4403.0 (12.9)	1367.4 (11.5)	3437.5 (12.9)
$100 000-$149 999	4871.8 (14.3)	1397.4 (11.7)	3803.2 (14.3)
$150 000 and over	3476.9 (10.2)	1064.1 (8.9)	2834.0 (10.6)
Did not respond	2057.3 (6.0)	856.5 (7.2)	1741.5 (6.5)
U.S. census division			
Northeast	7320.1 (21.5)	2611.9 (21.9)	6257.3 (23.5)
Midwest	9616.2 (28.2)	3111.3 (26.1)	7336.1 (27.5)
South	11 993.1 (35.2)	4404.8 (37.0)	9012.5 (33.8)
West	5176.6 (15.2)	1789.9 (15.0)	4076.6 (15.3)

### Sample of U.S. counties

**Figure 1:**
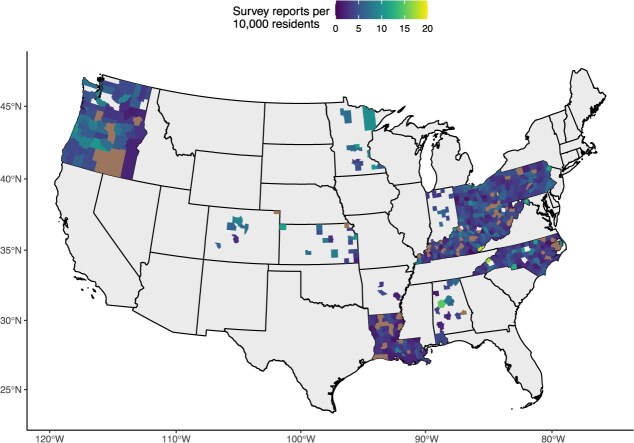
Map of masking reports from U.S. counties that issued mask mandates preceding, concurrent with, or in the absence of respective state mandate. U.S. respondents completed a web survey on their masking behavior between June 2, 2020, and January 1, 2021. Self-reports from the U.S. adults (*n* = 34 106) who lived in U.S. counties (*n* = 555) that issued mask mandates preceding, concurrent with, or in the absence of respective state mandates were included for analysis. Sixty-nine additional counties (*brown*) met the inclusion criteria for analysis but did not contribute masking self-reports in the 14 days premandate and/or postmandate issuance and were therefore excluded. County-level mandate information was extracted from the “Tracking Mask Mandates During the Covid-19 Pandemic” database.

Of the 624 counties eligible for analysis, 555 had at least one self-reported observation on mask-wearing both before and after their issuance of a mandate. Most mandates were implemented in July 2020 (*n* = 423 counties) compared to June 2020 (*n* = 132 counties). Of the 69 counties without sufficient reports, 20% were metropolitan compared to 47% of the included ones. The counties included were not evenly distributed geographically across the United States (Figure 1). Of the counties in the sample, the majority (*n* = 471) issued mandates on the same day as statewide mandates. In the 84 counties that issued mandates prior to statewide ones, the county mandates were issued a mean of 11.0 (SD: 6.5) days earlier. These counties were spread across 11 states, though the majority were in Ohio (*n* = 21), Indiana (*n* = 14), Alabama (*n* = 12), and Minnesota (*n* = 12).

### Masking in U.S. counties

The median number of completed surveys on masking per county was 18 [IQR: 8-51] surveys or 4.1 [IQR: 3.0-5.5] surveys per 10 000 residents of each included county. The total number of completed surveys (*N* = 16 569) prior to mandates was smaller than the number of completed surveys (*N* = 17 537) following mandates. Over time, there was a general trend of increased self-reported masking in public and private settings (Figure S1). The modeled probability to self-report masking in grocery stores went from 73.7% in the 2 weeks prior to mandates to 83.5% in the 2 weeks following their issuance. Reports of masking with family/friends went from 32.8% to 37.5% from the 2 weeks prior to mandates compared to the 2 weeks after.

### Effect on mask wearing in private settings

**Figure 2:**
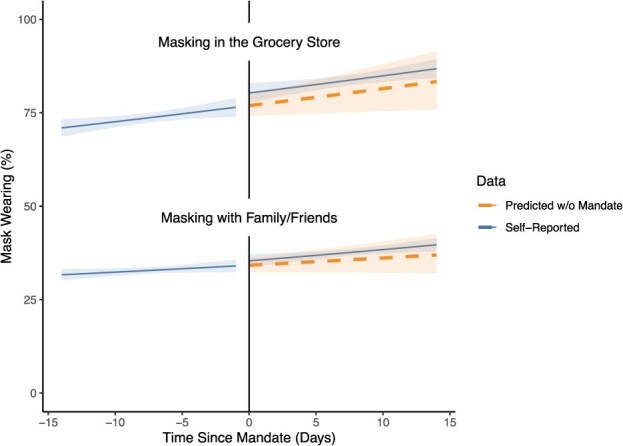
Self-reported masking in public and private settings pre- and post-mask mandate. U.S. respondents completed a web survey on their masking behavior between June 2, 2020, and January 1, 2021. Self-reports from the U.S. adults (*n* = 34 106) who lived in U.S. counties (*n* = 555) that issued mask mandates preceding, concurrent with, or in the absence of respective state mandates were included for analysis. Individuals were considered masked if they reported they were very likely to wear a mask “while grocery shopping” (masking in public settings) or “while visiting with family or friends in their homes” (masking in private settings). Stacked interrupted time series models were fit to self-reported masking (*blue*). Masking levels in the absence of a mandate (*orange*) were predicted from the models.

Stacked ITS analysis (Figure 2) of mask-wearing with family/friends found a very small pulse effect (RR: 1.03 [95% CI, 0.95-1.12]), and no ramp effect (RR: 1.00 [95% CI, 0.99-1.01]). This indicates that there may have been a minor one-time increase in masking but no change in private-setting masking behavior trends. The marginal estimates from this model suggest that, across all counties during the study period, the mean observed mask-wearing with family/friends was 1.1 percentage points [95% CI, −1.7 to 3.9] higher than what would have been expected if no mandates were in place. Similar findings for pulse (RR: 1.05 [95% CI, 0.96-1.15]) and ramp (RR: 1.01 [95% CI, 0.99-1.02]) effects were observed on pooled ITS sensitivity analysis.

### Effect on mask wearing in public settings

Stacked ITS analysis (Figure 2) found a small pulse effect (RR: 1.04 [95% CI, 1.01-1.08]) and no ramp effect (RR: 1.00 [95% CI, 1.00-1.00]) of mask-wearing in grocery stores following mandates. The marginal estimates from this model suggest that, across all counties during the study period, the mean observed mask-wearing in grocery stores was 3.4 percentage points [95% CI, 0.8-6.0] higher than what would have been expected if no mandates were in place. A sensitivity analysis utilizing a pooled ITS design and incorporating the effect of counties found similar pulse (RR: 1.03 [95% CI, 1.02-1.04]) and ramp (RR: 1.00 [95% CI, 1.00-1.00]) effects.

### County heterogeneity in the effect of mask mandates

**Figure 3:**
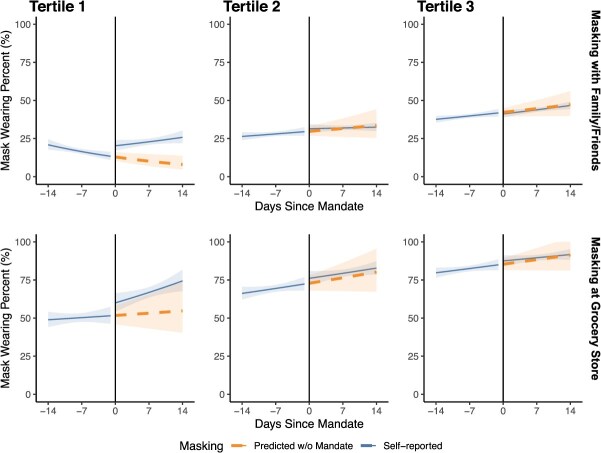
Self-reported masking in public and private settings pre- and post-mask mandate by county tertile of reported premandate masking. U.S. respondents completed a web survey on their masking behavior between June 2, 2020, and January 1, 2021. Self-reports from the U.S. adults (*n* = 34 106) who lived in U.S. counties (*n* = 555) that issued mask mandates preceding, concurrent with, or in the absence of respective state mandates were included for analysis. Individuals were considered masked if they reported they were very likely to wear a mask “while grocery shopping” (masking in public settings) or “while visiting with family or friends in their homes” (masking in private settings). Separate stacked interrupted time series models were fit to self-reported masking (*blue*) for counties based on the tertile of reported premandate masking levels. Masking levels in the absence of a mandate (*orange*) were predicted from the models. When computing the confidence intervals three upper bound estimates exceeded 1. These estimates were adjusted to 1 to reflect valid probabilities.

The effects of mandates on mask-wearing varied substantially across different counties (Table 2). In an analysis, where counties were stratified by masking levels prior to mandate implementation (Figure 3), the absolute effect of mandates was greatest in counties with the lowest prior masking levels and lowest in counties with high prior masking. In an analysis of geography, the absolute effect of mandates was greatest in counties in the U.S. West census region and lowest in counties in the Northeast region. In an analysis of urbanicity, the absolute effect of mandates was greatest in micropolitan and nonmetropolitan counties and lowest in metropolitan counties. In an analysis of the calendar time of when the mandates were issued, minimal difference was observed between counties that issued mandates in June compared to those issued in July. In geospatial adjacency analysis, we found that mandates were more effective if a neighboring county had issued a prior mask mandate.

**Table 2:** Results from stacked interrupted time series fit to self-reported masking levels pre- and post-mask mandate in U.S. counties (*n* = 555) across county characteristic and masking setting with masking defined as those very likely to mask.

**Mask wearing setting**	**County characteristic**	**Variable**	**Mean very likely to mask premandate, %**	**Mean very likely to mask postmandate, %**	**Estimated percentile increase of individuals likely masking attributed to mandate (95% CI)**
Grocery store	Prior masking	Lowest	50.2	67.0	8.0 (−0.6 to 16.5)
		Moderate	69.2	79.4	3.3 (−1.6 to 8.2)
		Highest	82.3	89.6	2.3 (−0.6 to 5.1)
					
	U.S. census region	South	69.4	81.1	3.5 (−1.1 to 8.0)
		West	72.4	86.0	7.5 (1.6-13.4)
		Midwest	70.6	82.3	2.2 (−2.6 to 7.0)
		Northeast	84.7	87.8	1.3 (−3.8 to 6.5)
					
	Mandate month	July	74.7	84.1	3.1 (0.1-6.1)
		June	71.2	81.9	3.4 (−1.5 to 8.4)
					
	Adjacent county prior mandate	Yes	70.5	81.2	2.7 (−2.8 to 8.2)
		No	74.5	84.2	3.7 (0.8-6.6)
					
	Urbanicity	Metro	76.0	85.0	3.0 (0.3-5.7)
		Nonmetro	58.7	71.4	5.6 (−9.0 to 20.3)
		Micropolitan	60.5	75.9	7.0 (−2.0 to 16.0)
With family and friends	Prior masking	Lowest	16.9	22.9	8.0 (0.7-15.3)
		Moderate	27.9	32.0	1.8 (−3.0 to 6.6)
		Highest	39.7	43.9	−1.0 (−4.9 to 2.8)
					
	U.S. census region	South	34.1	39.7	4.4 (−0.3 to 9.1)
		West	31.8	37.7	1.6 (−5.0 to 8.2)
		Midwest	29.4	35.5	0.3 (−4.8 to 5.4)
		Northeast	35.6	36.3	−4.0 (−10.8 to 2.7)
					
	Mandate month	July	32.8	36.9	0.6 (−2.8 to 3.9)
		June	32.9	38.8	2.8 (−2.3 to 7.9)
					
	Adjacent county prior mandate	Yes	30.0	34.8	−1.9 (−7.6 to 3.8)
		No	33.5	38.3	2.1 (−1.1 to 5.2)
					
	Urbanicity	Metro	33.8	38.8	0.9 (−2.1 to 4.0)
		Nonmetro	28.5	29.0	4.2 (−8.8 to 17.3)
		Micropolitan	26.2	29.7	2.0 (−6.4 to 10.3)

## Discussion

In this ITS estimating the effect of U.S. county-level mask mandates on self-reported mask-wearing, we find that mandates led to a modest (approximately 1%-3%) increase on self-reported masking in public and private settings. However, the effect of mandates on masking varied substantially (from approximately −4 to 8 percentage points) by geography, urbanicity, and prior masking levels.

The findings from our study help to clarify the effectiveness of mask mandates and to reconcile this with previous literature reporting mixed effectiveness. Our analysis suggests that mandates tended to have a slightly larger impact on self-reported masking in public compared to private settings. During the study period, mandates varied in their mechanism of enforcement. While some jurisdictions stipulated fines or penalties, in many cases they were primarily designed to exert social pressure and normalize mask-wearing in public settings. This distinction may explain why we observed a modest effect in grocery stores but a smaller change in self-reported household masking, where legal enforcement was impractical and social oversight weaker. However, in a prior modeling effort^5^ conducted during a single wave of the COVID-19 pandemic, it was estimated that a 1% increase in self-reported masking was associated with an approximately 13% higher odds of transmission control (R_t_ < 1 compared to R_t_ ≥ 1) suggesting that even modest improvements in masking may be meaningful for infection control; albeit heavily context dependent.

Our model also estimates a substantially larger impact of mandates in areas with low levels of self-reported mask usage prior to masking implementation. When mandates were implemented in these areas, the postmandate levels of mask usage were elevated to levels that were comparable to those seen in counties with higher self-reported mask usage before the mandates were put into effect. Given the heterogeneity of mandates in the United States, previous results suggesting mandates did not reduce transmission may be attributed to mandates being enacted in areas with already elevated masking behavior and a subsequently limited pool of unmasked individuals amenable to mandate-related behavioral changes. For example, we show here that in Northeast U.S. counties, where premandate masking behaviors were highest, masking may have only increased approximately 1-3 percentile points in the 2 weeks following mandates and our model ascribes almost none of this gain to the mandates themselves.

Prior estimates in the literature have found the effect of statewide mandates on masking behavior ranging from 23 percentage points to no change.^14^ Our estimates fall within this range and may be short of the largest estimated effect due to the fact that 3 (Iowa, North Dakota, and New Hampshire) of the 4 states in that analysis are largely rural and experienced a flat pretreatment masking trend, something not seen in the county-level data presented here. Studies that found no evidence of masking increases following mandates have aggregated data from states with large effects and null effects. They may also be subject to geographic spillover due to the fact some county mandates preceded statewide ones. Here, we found evidence to suggest an attenuation of a county’s mandate effectiveness when it is issued following an adjacent county’s mandate. State spillover effects may be even larger.

Importantly, the counties involved in this study (ie, those that issued mandates either prior to, in tandem with, or independent of their respective state mandates after June 17, 2020) represent only a small portion of the total counties in the United States. Given the substantial variability observed in the impact of mandates, these findings do not offer a definitive or comprehensive understanding of the effects of mandates broadly. However, the diversity of the counties included in this analysis does shed light on why previous studies may have yielded conflicting results. Specifically, we find the effect of mask mandates on masking is not uniformly consistent and supports the hypothesis that there is Effect Measure Modification by important county-specific factors such as the theoretical population pool of those willing to mask.

There are several additional limitations to the current analysis. Although the survey data has shown a high correlation with other sources, it is still reliant on anonymous self-reports that may reflect recall errors, social incentives related to masking, and the systematic exclusion of those with limited internet access. It is possible we are overestimating the effect of mandates because individuals believe our survey expects masking during mandated periods and therefore they are more likely to report masking (social desirability bias). Self-reports can also induce selection bias (eg, only those adhering to the law will want to report postmandate) that would bias estimates of mandate effectiveness away from the null. In addition, given the highly politicized context of COVID-19, it is plausible that respondents in certain regions may have systematically misreported their behaviors, either overstating compliance in areas supportive of mandates or overstating defiance in areas resistant to them (ie, preference falsification). Composition bias is also a concern, as respondents were SurveyMonkey users, a group likely skewed toward those who are more online and therefore also possibly more compliant with stay-at-home orders.

By utilizing counties, this analysis minimized urban/rural confounding found in statewide analyses but is still susceptible to bias created by other municipal mandates (eg, citywide ones) and spillover from changes introduced in nearby jurisdictions. Our model also assumes that there were no exogenous influences on masking behavior in the 2 weeks prior to the issuance of a mandate, which is unlikely to be true. Increases in masking may respond to the same disease pressures as mandates (eg, increases in cases). This confounding would cause our effect estimates to overreport the effect mandates would have on masking in isolation. However, this study still improves upon prior attempts as it directly measures the effect of mandates on masking and not mandates on disease outcomes, the latter of which is causally circular with pressures exerted by the disease itself, and therefore more susceptible to this bias. Individual-level studies would be ideal for disentangling the effect of policy on behavioral response, and the incorporation of this type of observational data into ongoing pandemic surveillance efforts is essential for designing evidence-backed intervention.

Notably, irrespective of the broad population effects of mandates on adherence, mandates may be an important tool to enforce norms that enable individual-level risk reduction and for especially vulnerable workers to protect themselves from infection (eg, citing mandates to enforce masking within a particular establishment). Mandates may also be an important tool to sustain previous increases in masking, which was not studied here. However, we have demonstrated an effect modification by important demographic characteristics in the effect of mandates on masking behavior, which may explain the conflicting effects of mandates on disease-related outcomes reported in the literature and provide helpful insight as to which regions may experience the greatest benefit of a mask mandate.

## Conclusion

Masking is an important tool to reduce respiratory disease transmission. Throughout 2020, mask mandates were introduced across U.S. states and counties to increase mask-wearing and combat the coronavirus pandemic. In this analysis, we showed that the effects of these mandates were substantially varied, and they only altered masking behavior in select contexts, such as when implemented when the current proportion of the population masking was low. Future pandemic response will need to consider that broad mandates may induce mixed effects and that crafting tailored and hyperlocal interventions may be a more efficient way to increase masking and reduce disease transmission.

## Supplementary Material

Web_Material_kwaf236

## Data Availability

Data requests can be sent to info@outbreaksnearme.org. Anonymous aggregated data are available in a dashboard for the public: https://public.tableau.com/app/profile/surveymonkey/viz/CovidNearYouSurveyMonkeyMasks/CNYSVMK.
