# Supplementary material for: Heterogeneous impact of mask mandates on U.S. masking behavior: an interrupted time series study
Source: Am J Epidemiol. 2025 Oct 22;195(2):488–96. doi: 10.1093/aje/kwaf236 (PMC13368596; doi:10.1093/aje/kwaf236)
Supplement: Web_Material_kwaf236 [file web_material_kwaf236.zip › Mask_Mandates_supplement_10_16_25.docx]

**SUPPLEMENTARY MATERIALS**

Heterogeneous Impact of Mask Mandates on U.S. Masking Behavior: An Interrupted Time Series Study

Benjamin Rader, Christina M. Astley, Laura F. White, John S. Brownstein, Matthew P. Fox

CONTENTS

APPENDIX S1 Sensitivity Analysis of Model Specification

FIGURE S1 Self-reported masking in public and private settings over time

TABLE S1 Self-reported demographics and masking behavior from U.S. adults (n=34,106) who lived in U.S. counties (n=555) that issued mask mandates preceding, concurrent with, or in the absence of respective state mandates, by mandate status at date of survey collection.

REFERENCES S1

APPENDIX S1 Sensitivity Analysis of Model Specification

To ensure our results were robust to model specification and ITS parameterization, we conducted a sensitivity analysis utilizing a pooled design ITS model. The pooled design fits separate ITS models for each county ($j= 1, 2, .., k$) and pools parameter estimates to measure the effect of mandates. While the pooled design is well suited to capture the effect of mandates across many counties, it produces unstable parameter estimates in counties with just a few survey observations, a problem the stacked design is less vulnerable to^1^. Of the 555 counties that met the inclusion criteria, the median [IQR] respondents was 18 [8-51] (which we have added to the manuscript).The pooled ITS design also utilized a Poisson regression model with a log link function and robust error variance:

$ln[{p(Masking)}_{s,j}]= \beta_{0}+ \beta_{1}Time_{j}+ \beta_{2}Mandate_{j}+\beta_{3}TimeSinceMandate_{j}$.

To estimate the general effect of mask mandates across all counties, pooled parameter estimates were calculated by an inverse variance weighted average of each of the individual model parameter coefficients ($n=1,2,3$)^1^:

$$\beta_{n, pooled}= \frac{\sum_{j=1}^{k} w_{n,j}\beta_{n,j}}{\sum_{j=1}^{k} w_{n,j}},$$

where $w_{n,j}= \frac{1}{{(Standard Error\left[ \beta_{n,j} \right])}^{2}}$ and the pooled variance is: ${var(\beta}_{n, pooled})=\frac{1}{\sum_{j=1}^{k} w_{n,j}}$. Wald-based confidence intervals are then constructed for each parameter $\beta_{n, pooled}\pm1.96\sqrt{{var(\beta}_{n, pooled})}.$

FIGURE S1 Self-reported masking in public and private settings over time


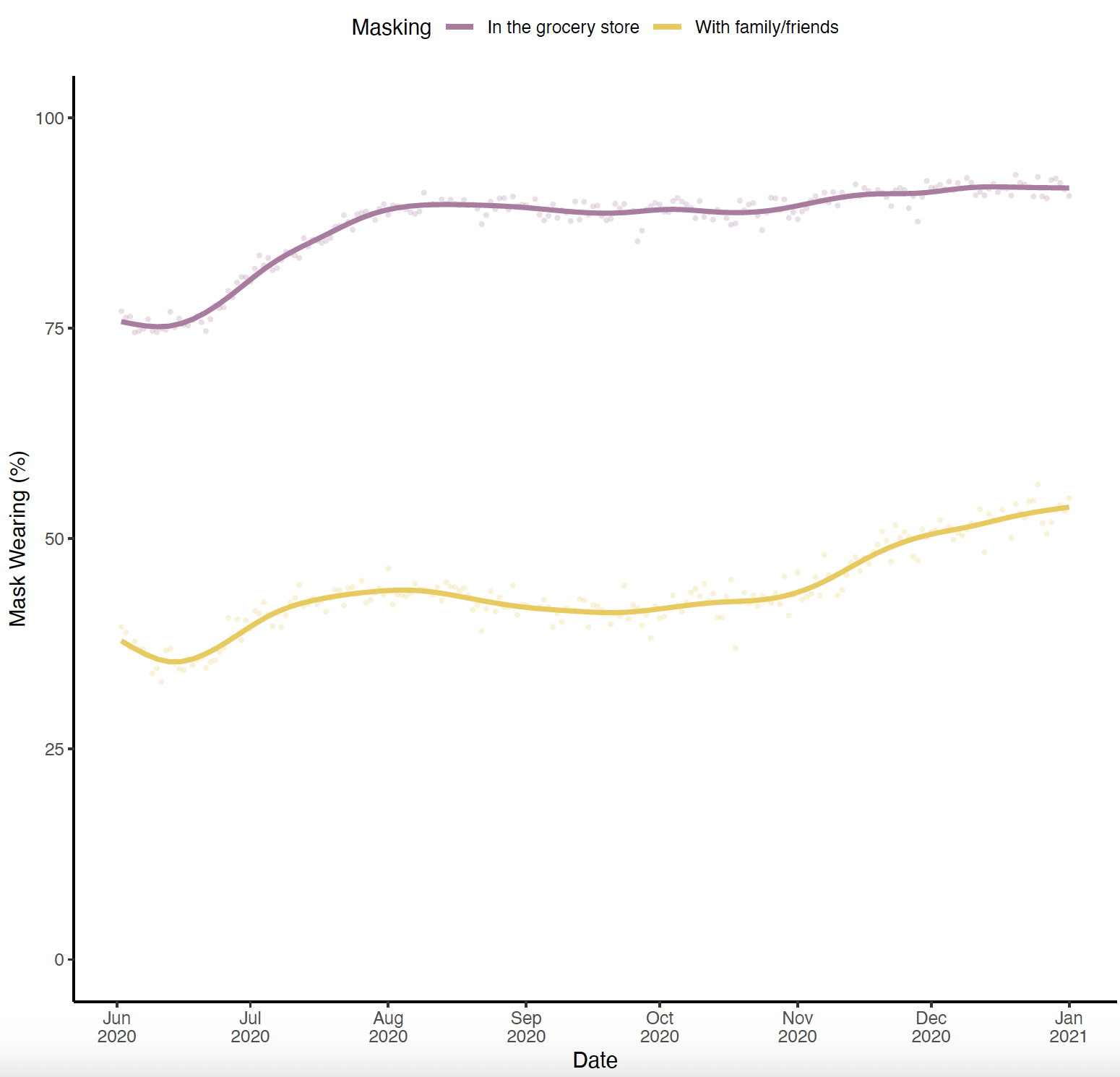


Survey weighted proportions of respondents reporting they were “very likely” to wear a mask in public (grocery store) or private (with family/friends) settings between June 2, 2020 and January 1, 2021. Smooth trends were estimated using generalized additive models (cubic regression splines) applied to daily data.

TABLE S1 Self-reported demographics and masking behavior from U.S. adults (n=34,106) who lived in U.S. counties (n=555) that issued mask mandates preceding, concurrent with, or in the absence of respective state mandates, by mandate status at date of survey collection.

| **Characteristic** | **Pre-Mandate Survey Respondents, N (%)** | **Post-Mandate Survey Respondents, N (%)** |
| --- | --- | --- |
| Race |  |  |
| White | 13102 (7.0) | 13785 (7.4) |
| Black | 1882 (1.0) | 1997 (1.1) |
| Hispanic | 361 (0.2) | 391 (0.2) |
| Other | 524 (0.3) | 541 (0.3) |
| Missing | 700 (0.4) | 823 (0.4) |
| Gender |  |  |
| Female | 11012 (5.9) | 11718 (6.3) |
| Male | 5370 (2.9) | 5613 (3.0) |
| Transgender or Nonbinary | 187 (0.1) | 206 (0.1) |
| Education |  |  |
| High School or Less | 2620 (1.4) | 2729 (1.5) |
| Some College | 4650 (2.5) | 4941 (2.6) |
| College or More | 4953 (2.6) | 5284 (2.8) |
| Post Graduate Degree | 4346 (2.3) | 4583 (2.5) |
| Age |  |  |
| 18-29 years | 1601 (0.9) | 1884 (1.0) |
| 30-39 years | 2567 (1.4) | 2881 (1.5) |
| 40-49 years | 3358 (1.8) | 3615 (1.9) |
| 50-64 years | 5528 (3.0) | 5843 (3.1) |
| 65-74 years | 2608 (1.4) | 2498 (1.3) |
| 75+ years | 907 (0.5) | 816 (0.4) |
| Household Income |  |  |
| Less than $30,000 | 2672 (1.4) | 2883 (1.5) |
| $30,000-49,999 | 2173 (1.2) | 2452 (1.3) |
| $50,000-$74,999 | 2729 (1.5) | 2819 (1.5) |
| 75,000-$99,999 | 2435 (1.3) | 2472 (1.3) |
| $100,000-$149,999 | 3036 (1.6) | 3179 (1.7) |
| $150,000 and Over | 2514 (1.3) | 2674 (1.4) |
| Did Not Respond | 1010 (0.5) | 1058 (0.6) |

REFERENCES S1

1. Gebski V, Ellingson K, Edwards J, Jernigan J, Kleinbaum D. Modelling interrupted time series to evaluate prevention and control of infection in healthcare. *Epidemiology and Infection*. 2012;140(12):2131-2141. doi:10.1017/S0950268812000179
